# Interactions between Human Serum Albumin and Sulfadimethoxine Determined Using Spectroscopy and Molecular Docking

**DOI:** 10.3390/molecules27051526

**Published:** 2022-02-24

**Authors:** Yuai Zhang, Yiqing Cao, Yan Li, Xuemei Zhang

**Affiliations:** 1 Department of Pharmacology, School of Pharmacy, Fudan University, Shanghai 201203, China; 20111030019@fudan.edu.cn; 2 NanChang Bozekang Pharmaceutical Technology Co., Ltd., Nanchang 330000, China; 3 Department of Pharmaceutical Analysis, School of Pharmacy, Fudan University, Shanghai 201203, China; cpucyq@163.com

**Keywords:** sulfadimethoxine, molecular docking, spectroscopy, human serum albumin

## Abstract

Sulfonamides are widely used antibiotics in agricultural production. However, the potential threat of these drugs to human health has increased global concern. Human serum albumin (HSA) is the main reservoir and transporter of exogenous small molecules in humans. In this study, the interaction between sulfadimethoxine (SMT) and human serum albumin (HSA) was studied using spectroscopy and computer simulation. Our results showed that the hydrogen bonding and van der Waals forces drove SMT to enter the binding site I of HSA spontaneously and resulted in the fluorescence quenching of HSA. The stability of the HSA–SMT complex decreased with an increase in temperature. The binding of SMT to HSA induced alterations in the secondary structure of HSA, where the content of α-helix decreased from 61.0% of the free state to 59.0% of the compound state. The π–π, π–σ, and π–alkyl interactions between HSA and SMT were found to play important roles in maintaining the stability of the complex.

## 1. Introduction

As one of the most widely used groups of antimicrobials, sulfonamides, including sulfadimethoxine (SMT), play a pivotal role in agriculture [1]. However, recent studies have shown that sulfonamides, together with their metabolites, may pose a potential threat to human health by virtue of their accumulation in the body through the food chain [2]. For example, Dimitrios B et al. found that sulfonamides not only induce drug-resistant bacteria in human intestinal flora but also lead to abnormalities including those of the liver and kidneys [3]. To safeguard public health, the permissible limits of sulfonamides in food (e.g., bread, eggs, and milk) and feed established by the EU, US-FDA, and China are 100 μg/kg [4]. Unfortunately, sulfonamides are a growing cause of health concern because of its high levels in edible agricultural and food products [5,6]. Therefore, there is an urgent need to determine the mechanism of toxicity of sulfonamides on human health.

Human serum albumin (HSA), the most abundant protein in plasma, is quite versatile in function, and plays a role in maintaining the acid–base balance, regulating osmotic pressure, and catalyzing metabolic reactions [7,8]. In addition, HSA is the primary depot and is involved in the transport of exogenous small molecules throughout the body [9,10], which is, consequently, accompanied by conformational changes in its secondary and tertiary structures. On the other hand, it is well known that the functions of proteins are closely related to their spatial orientation and configuration [11]. Due to the physiological functions of HSA, the interaction between drug molecules and HSA is currently the focus of research [12]. The HSA molecule is divided into three structural domains (I, II, III), each of which contains two subdomains (IA, IB, IIA, IIB, IIIA, IIIB). HSA has two main drug sites consisting of hydrophobic cavities (site I and site II), site I is located in subdomain IIA; site II is located in subdomain IIIA. The structure of HSA has been determined by various analytical techniques, including fluorescence spectrometry, circular dichroism (CD), nuclear magnetic resonance, electrochemistry, equilibrium dialysis, and molecular modeling [13,14]. Among them, fluorescence spectroscopy is a fast and sensitive method, which can obtain a great deal of relevant information of drug–HSA complexes, such as the binding constant, binding forces, quenching mechanism, and the conformational change in HSA caused by drug molecules [15]. Therefore, studying the binding mechanism of drugs to HSA is a prerequisite to understanding the effects of drugs in humans.

The multivariate curve resolution-alternating least squares (MCR-ALS) method, which can be used to simultaneously analyze multiple data matrices obtained by different experimental techniques or bidirectional experimental methods [16,17], provides a simple and efficient soft modeling method for solving the problem of signal overlap problem [18]. Furthermore, we can acquire some key information through the results of the MCR-ALS, including the number of individual chemicals and their pure spectral and concentration profiles [19]. Therefore, the MCR-ALS method is of great benefit to understanding the mechanism of binding SMT with HSA.

As we all know, computer simulation has become the most widely used method for describing the binding models of drug molecules and proteins. Detailed and useful ligand–protein complex parameters can be obtained by molecular docking [20].

Among sulfonamides, sulfadimethoxine (SMT) plays an indispensable role and has the advantages of a wide antibacterial spectrum, stability, and ease of use. However, it is difficult to eliminate this compound from the environment [21]. Furthermore, sulfamethoxine is an antibiotic drug widely used in agricultural production, which can directly act on human beings through the food chain, so its potential threat to human health has attracted global attention. The interaction between sulfonamides and HSA can cause changes in the microenvironment and conformation of HSA. By studying the combination of SMT and HSA, we can understand the drug metabolism process of SMT in vivo. Spectroscopic methods show that sulfonamides can spontaneously enter the binding site I of HSA through hydrogen bonds and van der Waals forces. Like sulfadimethoxine that binds to HSA at position I, sulfonamides that also bind to HSA at position I include sulfamethoxine (SMD) and sulfamonomethoxine (SMM). To the best of our knowledge, there are only three studies that report the binding of SMT and HSA, and SMT and bovine serum albumin (BSA). Otagiri et al. used CD, fluorescence, and dialysis techniques to determine the binding of SMT and N4-AcSMT to HSA and rabbit serum albumin [22,23]; however, their study did not address the key factors that drive the binding of the two [24].

In this study, a simple and efficient method was developed based on spectroscopy and molecular docking to explore the binding mechanism of SMT and HSA. Specifically, we used thermodynamics to determine the driving forces between SMT and HSA, followed by infrared spectroscopy to study the effect of SMT on the conformation of HSA. Lastly, the mode of binding of SMT and HSA was determined using molecular docking. The experimental results showed that SMT and HSA bind to site I, which was consistent with the molecular docking simulation results.

## 2. Results

### 2.1. Determination of the Interaction Pattern between SMT and HSA

**Figure 1:**
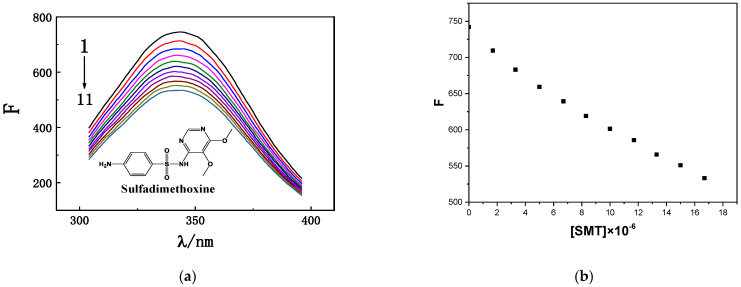
(**a**) Fluorescence emission spectra of HSA with different SMT concentrations. C_HSA_ = 1.7 × 10^−6^ mol·L^−1^; C_SMT_ (1–11) = 0, 1.7, 3.3, 5.0, 6.7, 8.3, 10.0, 11.7, 13.3, 15.0, 16.7 × 10^−6^ mol·L^−1^. (**b**) Fluorescence intensity corresponding to different SMT concentrations.

HSA has a characteristic fluorescence peak at approximately 350 nm [24]. When SMT was added to HSA solution, the fluorescence intensity of HSA was quenched, and its position exhibited a blue shift toward 338 nm. As an important component of protein sequences, tryptophan (Trp), tyrosine (Tyr), and phenylalanine (Phe) emit a certain intensity of endogenous fluorescence [25]; accordingly, fluorescence-quenching experiments are often used to determine the interactions between exogenous small molecules and biological macromolecules. As shown in Figure 1, when the concentration of HSA in the study system was fixed, its fluorescence intensity decreased significantly when the concentration of SMT was increased from 0 to 16.7 × 10^−6^ mol·L^−1^, indicating that SMT quenched the endogenous fluorescence of HSA, i.e., there was an interaction between SMT and HSA [26].

**Figure 2:**
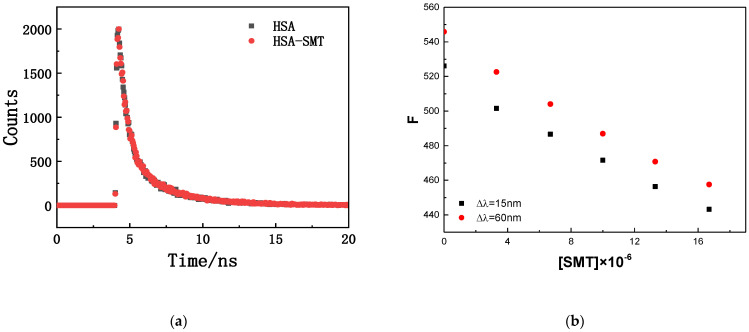
(**a**) Fluorescence decay curve of HSA in the absence and presence of SMT. C_HSA_ = C_SMT_ = 3.3 × 10^−6^ mol·L^−1^. (**b**) Fluorescence intensity corresponding to different SMT concentrations.

The fluorescence-quenching mechanisms of biomacromolecules can be divided into the following two types: dynamic quenching caused by molecular collision and static quenching caused by complex formation. The specific quenching type can be determined by the effect of exogenous substances on the fluorescence lifetime of the biomacromolecule. As demonstrated in Figure 2, there was an overlap in the time-resolved fluorescence spectra of HSA in the absence and presence of SMT, and the results were in accordance with the double exponential decay model (the values of two were close to 1.00). The fluorescence lifetime of free and composite HSA was 5.93 ns and 5.90 ns, respectively, which meant that there were no differences between them. Static quenching does not affect the fluorescence lifetime of biological macromolecules, whereas dynamic quenching is just the opposite [27]. Thus, the interaction between HSA and SMT could be attributed to static quenching, combined with the data of steady-state and transient fluorescence.

### 2.2. Thermodynamic Experiments

**Figure 3:**
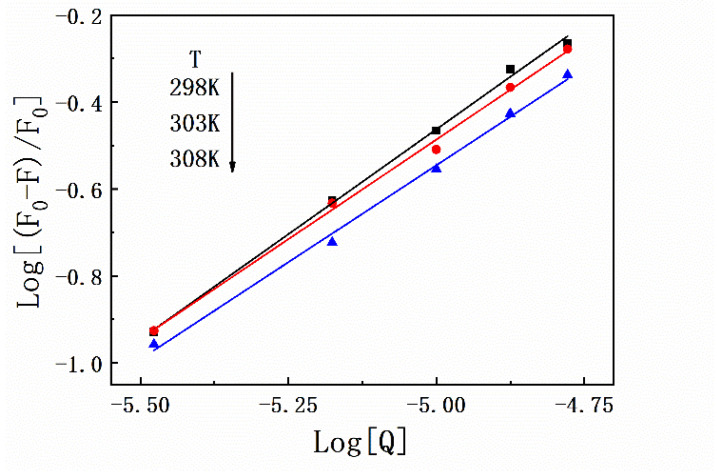
Plots of Log[(F0 − F)/F0] versus Log[Q] for HSA–SMT complex under different temperatures.

Thermodynamic experiments are a classic method to determine the types of forces between protein–ligand complexes based on the theory proposed by Ross. Briefly, the interaction between exogenous small molecular substances and biological macromolecules can usually be determined on the basis of the thermodynamic constants (ΔG, ΔS, and ΔH). In this study, fluorescence quenching of HSA by SMT was performed at 298 K, 303 K, and 308 K, and the corresponding binding constants were 2.31 × 10^−4^ L·mol^−1^, 1.25 × 10^−4^ L·mol^−1^, and 0.83 × 10^−4^ L·mol^−1^, respectively. These results indicated that the stability of HSA–SMT was inversely regulated by temperature, which in turn supported the conclusion that SMT led to the static quenching of HSA [28]. As listed in Table 1, the detailed data of thermodynamic parameters were calculated using Equations (3) and (4). As demonstrated in Figure 3, the number of binding sites always tended to 1 at different temperatures, indicating that SMT had a single binding site on HSA. The negative ΔG suggests that the binding of SMT to HSA was spontaneous, while the negative ΔS and ΔH indicate that van der Waals force and hydrogen bonding are the main driving forces in the binding of SMT to HSA.

### 2.3. Effect of SMT on the Conformation of HSA

**Figure 4:**
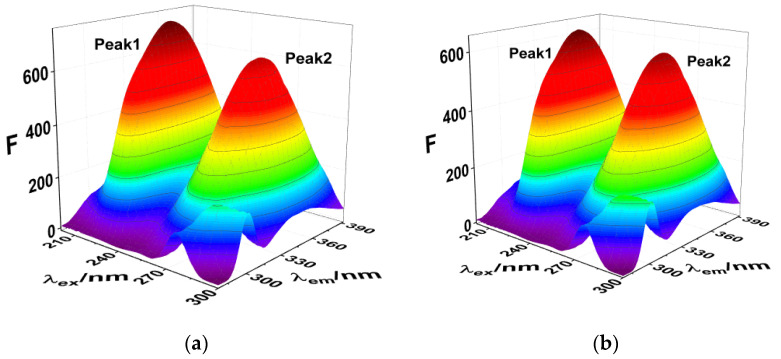
(**a**) 3D fluorescence spectra of HSA; (**b**) 3D fluorescence spectra of HSA–SMT. C_HSA_ = C_SMT_ = 3.3 × 10^−6^ mol·L^−1^.

Three-dimensional fluorescence spectroscopy and infrared spectroscopy are used to intuitively determine the influence of exogenous small molecules on the secondary structure of proteins. The influence of SMT is shown in the three-dimensional fluorescence spectrum of HSA in Figure 4 and Table 2, where Peak 1 is related to the peptide skeleton structure of HSA and Peak 2 represents the fluorescence characteristic peak of the aromatic amino acid residues. Upon combining with SMT, the intensity of the two fluorescence peaks of both free and bound HSA decreased significantly, indicating an interaction between SMT and HSA. Furthermore, a decrease in the fluorescence intensity of Peak 2 meant that SMT entered the hydrophobic cavity of HSA [29], whereas the stoke displacement of Peak1 decreased from 112 nm to 109 nm, suggesting that the presence of SMT induced changes in the secondary conformation of HSA.

**Figure 5:**
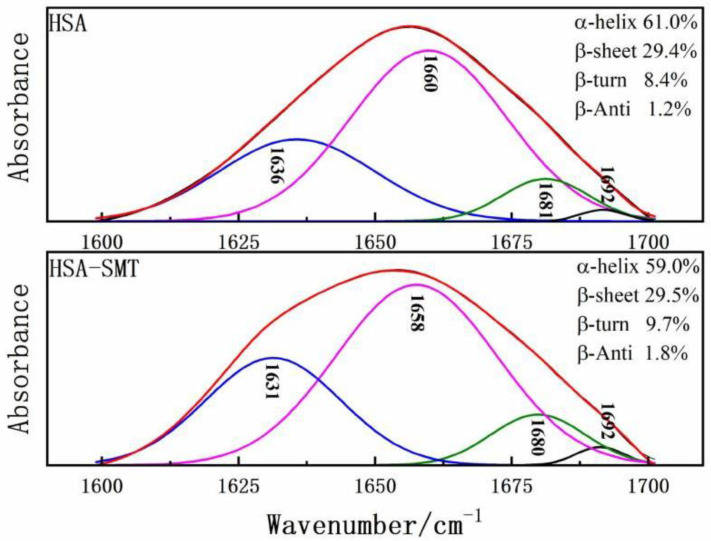
Curve-fit for HSA and HSA–SMT in the amide Ι region from 1700–1600 cm^−1^.

FT-IR was performed to further obtain detailed information on the changes of the secondary structures of HSA. HSA has many characteristic absorption bands in the infrared spectrum, among which the amide I band of 1600–1700 cm^−1^ is most sensitive to changes in the secondary structure [30]. Therefore, to determine the influence of SMT on the content of each secondary structure of HSA, baseline correction, deconvolution, and second derivative fitting were used to quantitatively analyze the data of the amide I band. Upon combining with SMT, the maximum peak position of the amide I band of HSA shifted to the direction of the short wave number, and the secondary structure of the composite HSA changed. As demonstrated in Figure 5, HSA-SMT compare with free HSA, the content of α-helix decreased from 61% to 59%, which indicated that the physiological function of HSA may be affected in vivo, as well as the distribution, transport, and metabolism of SMT. Besides, the presence of SMT led to changes in the secondary structure of HSA [31].

### 2.4. Binding Site

**Figure 6:**
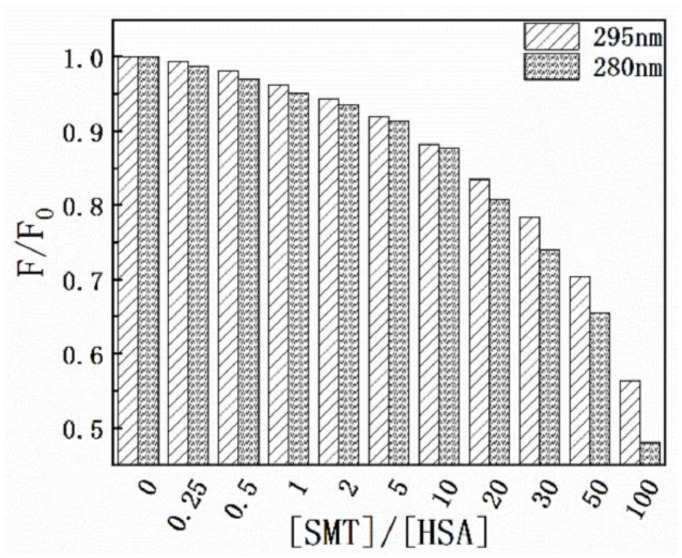
Effect of SMT on HSA fluorescence intensity at λ_ex_ = 280 nm and λ_ex_ = 295 nm.

**Figure 7:**
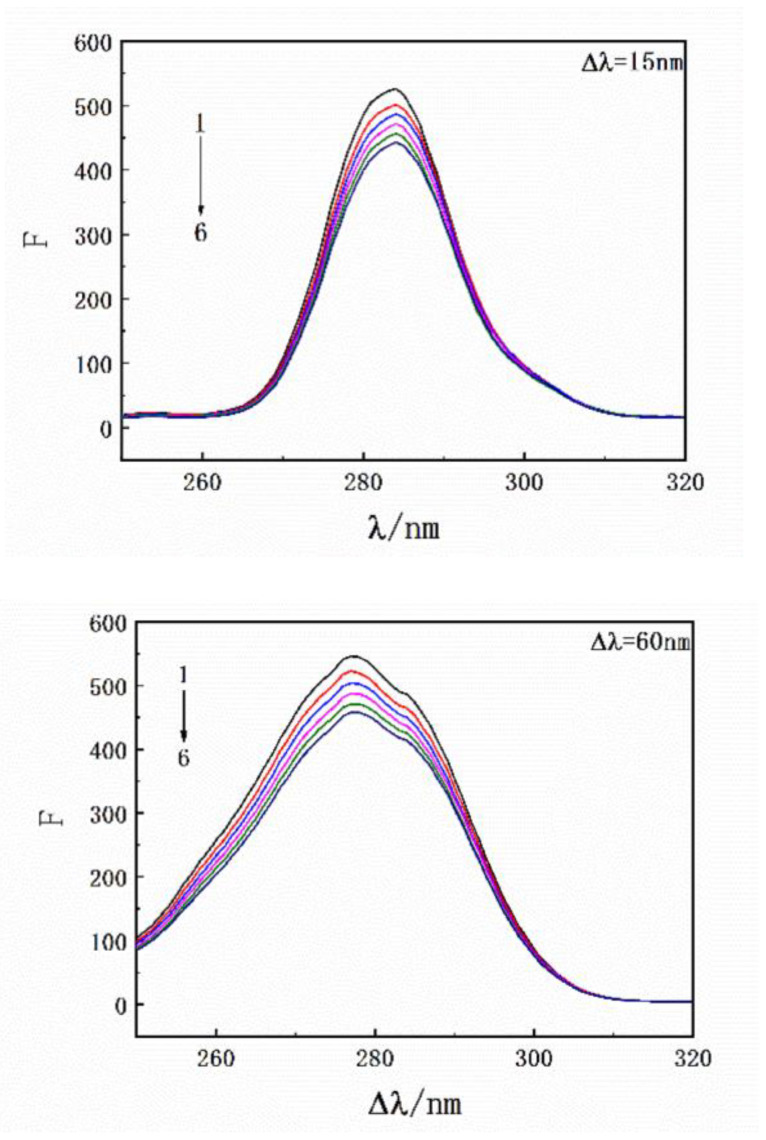
Synchronous fluorescence spectroscopy of HSA with an increase in drug concentration. C_HSA_ = 3.3 × 10^−6^ mol·L^−1^; C_SMT_ = 0, 3.3, 6.7, 10.0, 13.3, 16.7 × 10^−6^ mol·L^−1^.

Results of the thermodynamic experiments demonstrated that there was only one binding site between HSA and SMT. In general, for small molecules, HSA has two binding sites located in the hydrophobic cavity, namely sites I and II. The endogenous fluorescence of the protein is mainly derived from the two aromatic amino acid residues, Tyr and Trp, where Tyr exists in the two binding sites and Trp only exists in site I. Hence, the difference in fluorescence characteristics between Trp and Tyr was studied and used to explore the binding site between SMT and HSA [32]. The influence of SMT on HSA fluorescence intensity was tested at different excitation wavelengths (280 nm and 295 nm). Figure 6 shows that SMT quenched the endogenous fluorescence of HSA to a greater extent when the excitation wavelength was 280 nm, indicating that the presence of SMT affected the fluorescence of Tyr and Trp simultaneously. As demonstrated in Figure 7, the fluorescence intensity of Tyr and Trp decreased with an increase in SMT concentration, suggesting that these two amino acid residues were involved in the binding of HSA and SMT. Collectively, our results indicated that site I was the binding site for SMT on HSA. Furthermore, the presence of SMT had an impact on the microenvironment of Tyr because there was a small degree of redshift at the maximum emission wavelength.

### 2.5. Molecular Docking

**Figure 8:**
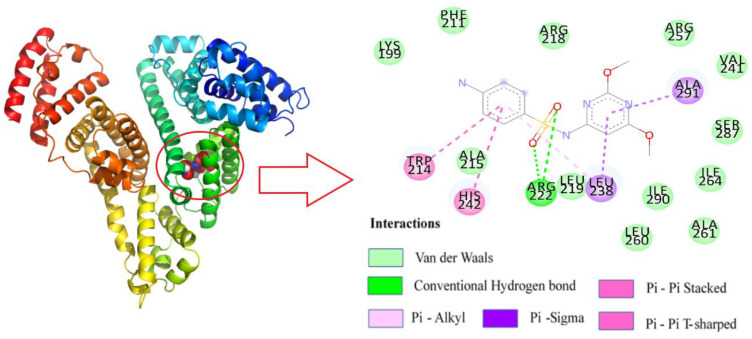
Molecular docking of HSA with SM.

After determining that SMT binds to site I of HSA, based on the binding-site studies, Autodock Vina was used to construct the three-dimensional conformation when the two were combined in a 1:1 ratio (Figure 8). The result indicated that SMT could spontaneously enter the hydrophobic cavity of HSA. The combined Gibbs free energy of SMT and HSA was determined to be −6.6 Kcal/mol. Further analysis using Discovery Studio 2018 software showed that SMT was surrounded by a few amino acid residues, including Trp214, Arg222, Lys199, Val241, Ala261, and Leu260, upon entering the hydrophobic cavity. Among them, van der Waals force was predominant and there were two hydrogen-bonding interactions between Arg222 and the two oxygen atoms on the sulfa group, which was consistent with the prediction from the thermodynamic experiments. Moreover, the π–π forces between the benzene rings of SMT and the Trp214 and His242 residues of HSA, and the π–σ and π–alkyl forces between Ala219 and Leu238 and SMT were also found to be present. The π–π interaction between the π electrons of SMT and Trp214 was the main factor leading to the quenching of the fluorescence of HSA. These forces played a role in conferring stability to the HSA–SMT structure.

Because HSA has many characteristic absorption bands in the infrared spectrum, especially the amide I band of 1600–1700 cm^−1^ which is the most sensitive to changes in the secondary structure. The existence of SMT leads to the movement of the maximum peak of HSA amide I band to the direction of short wave number, and the secondary structure of the composite HSA changes. Moreover, these small differences can cause structural changes. Through a series of experiments, the binding site I of SMT and HSA was determined. According to the results of Autodock Vina software, SMT could spontaneously enter the hydrophobic cavity of HSA, and the Gibbs free energy of the combination of the two was −6.6 Kcal/mol. Therefore, the slight difference was meaningful and similar to many published studies.

Moreover, infrared data of amide I band in HSA and HSA–SMT systems were analyzed by baseline correction, deconvolution, and second derivative fitting techniques, and their two-dimensional structure proportion analysis was shown in Table 3:

## 3. Discussion

In this study, we used several techniques to explore the binding mechanism between SMT and HSA. Site experiments revealed that SMT was bound to site I of HSA and formed a stable complex. Thermodynamic studies revealed that the stability of the complex was regulated by temperature. Results from the molecular docking study indicated that apart from van der Waals forces and hydrogen-bonding interactions, alkyl and π–σ forces played an important role in binding and conferring stability. Overall, our findings provide important biophysical insights into the potential threats of SMT to human health.

Our experimental results were different and similar to the published studies. Through fluorescence quenching experiments, circular dichroism, synchronization, and three-dimensional fluorescence spectroscopy experiments, Ma et al. [33] found that N-trans caffeine binds to HSA, and the binding region was at site I of the IIA subdomain of HSA. This result was consistent with our molecular docking computer simulation results. Gan et al. found that tragliptin (TLP) binds to HSA through fluorescence quenching experiments, calculation of binding constants and thermodynamic analysis. Synchronous fluorescence and three-dimensional fluorescence experiments showed that TLP had an effect on the microenvironment of amino residues, and circular dichromatogram analysis showed that TLP could bind to HSA and the binding region was at position I of the IIA subdomain of HSA, which was similar to our molecular dynamics simulation. Liao et al. [34] calculated the binding constants and thermodynamic analysis by fluorescence quenching experiment and found that SMD and SMM could bind to HSA. Moreover, a synchronous fluorescence experiment, three-dimensional fluorescence experiment, and circular dichromatographic analysis showed that SMD and SMM changed the secondary structure of HSA and determined that the binding region was at site I of the IIA subdomain of HSA, which was consistent with our experimental results again.

In conclusion, our experiment found that SMT could bind to HSA, and the binding not only affected the secondary structure of HSA, but also led to a decrease in the concentration of SMT in the blood, which provided a new direction for the study of SMT in human blood concentration.

## 4. Materials and Methods

### 4.1. Chemicals and Apparatus

Agilent Cary Eclipse fluorescence spectrophotometer with temperature control system for steady-state fluorescence detection, Quantaurus-Tau C11367-11(Hamamatsu Photonics, Hamamatsu, Japan) used for fluorescence lifetime detection. UV spectra were obtained using UV5500PC UV-Vis Spectrophotometer ((Shanghai metash instruments Co.,Ltd., Shanghai, China). The pH of the preparation of buffer solution was adjusted, used for a Mettler Toledo EL20 pH meter (Zurich, Switzerland).

Sulfadimethoxine (CAS:122-11-2) (4-amino-N-(2,6-dimethoxy-4-pyrimidinyl) benzenesulfonamide, HSA, and trihydroxyaminomethane (Tris) were purchased from Shanghai Yuanye Biotechnology Co., Ltd. (Shanghai, China). All other reagents were of analytical grade, and ultrapure water was used for all experiments. To prepare 0.05 mol·L^−1^ Tris-HCl buffer (containing 0.15 mol·L^−1^ NaCl), 1.2 g of Tris and 1.8 g of NaCl were dissolved in 200 mL of ultrapure water and the pH was adjusted to 7.4 using 18% HCl. The stock standard solution of HSA (1 × 10^−3^ mol·L^−1^) was prepared in Tris-HCl buffer and stored at −20 °C until further use. The stock standard solution of SMT (1 × 10^−1^ mol·L^−1^) was prepared in methanol, and a series of standard solutions were obtained by serially diluting the standard stock solutions with Tris-HCl buffer.

### 4.2. Methods

Fluorescence spectroscopy was recorded using a Cary Eclipse fluorescence spectrophotometer (Agilent, Santa Clara, CA, USA) equipped with a single-sample pool air-cooled Peltier attachment. Emission spectra were measured from 300–400 nm at 298, 303, and 308 K by setting the excitation wavelength to 280 nm. Considering the inner-filter effect of small molecular ligands, all steady-state fluorescence intensities in this study were corrected using the following equation [35]:

(1) $$F_{\mathrm{cor}}=F_{\mathrm{obs}}\times e^{\frac{A_{\mathrm{ex}}+A_{\mathrm{em}}}{2}}$$

where F_cor_ and F_obs_ are the corrected and observed fluorescence intensities, respectively, and A_ex_ and A_em_ are the absorbances at the excitation and emission wavelengths, respectively.

#### 4.2.1. Steady-State Fluorescence Spectroscopy

The concentration of HSA was maintained at 1.67 × 10^−6^ mol·L^−1^, and that of SMT was increased successively by 1 × 10^−6^ mol·L^−1^. Next, the fluorescence and synchronous fluorescence spectra were scanned and recorded using appropriate instrument parameters. The excitation and emission wavelength ranged from 200–300 nm and 280–390 nm, respectively, and the three-dimensional luminescence spectra of HSA and the HSA–SMT complex were acquired by increasing the excitation wavelength by 1 nm.

#### 4.2.2. Time-Resolved Fluorescence Spectroscopy

Time-resolved fluorescence was obtained for HSA and HSA–SMT mixtures at 280 nm as the excitation wavelength and 340 nm as the emission wavelength. The fluorescence lifetime of HSA was recorded when the count peak was 2000. Lastly, U11487, an instrument-specific software for data fitting, was used to calculate the average fluorescence lifetime.

#### 4.2.3. Thermodynamic Studies and Determination of the Binding Constant

The effect of SMT on the fluorescence of HSA was investigated at system temperatures of 298 K, 303 K, and 308 K. The binding constant (K_a_) of SMT and HSA, free energy (ΔG), entropy change (ΔS), and enthalpy change (ΔH) were determined using the equations listed below. Specifically, K_a_ was calculated using Equation (2), and the thermodynamic constant of the HSA–SMT system was obtained by substituting K_a_ into Equations (3) and (4).

(2) $$\log\frac{F_{0}-F}{F}=\log K_{a}+n\log[\mathrm{SMT}]$$

(3) $${\mathrm{nK}}_{a}=-\frac{\Delta H}{\mathrm{RT}}+\frac{\Delta S}{R}$$

(4) ΔG=ΔH−TΔS

where F_0_ and F represent the fluorescence intensities of the biomolecule with and without the quencher, respectively; n is the number of binding sites between SMT and HSA; and R is the gas constant (8.314 J·mol^−1^·K^−1^).

#### 4.2.4. Infrared Spectroscopy

The IR spectra of HSA and the HSA–SMT complex were recorded in the range of 1700–1600 cm^−1^ at 25 °C. The spectral data obtained by a resolution of 4 cm^−1^ and 64 scans were averaged. Subsequently, Fourier deconvolution and second derivative were used to analyze the percentage of HSA secondary structures. With the ATR attachment, origin was used to separate the small peaks in it, and deconvolution was carried out after 32 repetitions. The sample was in solution state.

#### 4.2.5. Molecular Docking

For docking simulation, the 3D ligand structure of SMT (ZINC: 13233295) was obtained from the Zinc15 small molecule database. The 3D crystal structure of HSA (PDB: 1H9Z) was downloaded from the RCSB protein data bank. DeepView was used to check the integrity of the software tutorial and USES default parameters. The coordinates of the docking center of the crystal structure of HSA supplemented the missing amino acid residues at the end. The crystal structure of the HSA–SMT complex was obtained using Autodock Vina, where the docking parameters were as follows: x, y, and z were 32.8, 13.5, and 9.6, respectively. The size of the box was 10, 10, and 10 with a resolution of 1Å. Lastly, the conformer with the lowest binding energy was selected as the most probable binding conformation and shown using Pymol and Discovery Studio 2018 Client.

## Figures and Tables

**Table 1:** Thermodynamic constants of interaction between SMT and HSA at different temperatures.

Temperature (K)	n	K_a_ (L·mol^−^^1^)	R	ΔH (kJ·mol^−^^1^)	ΔS (J·K^−^^1^·mol^−^^1^)	ΔG (kJ·moL^−^^1^)
298	0.9654	2.31 × 10^4^	0.997	−52.33	−90.91	−24.89
303	0.9167	1.25 × 10^4^	0.992			−24.16
308	0.8924	0.83 × 10^4^	0.965			−23.47

**Table 2:** Characteristic parameters of 3D fluorescence spectra.

	Peak	Peak Position λex/λem (nm/nm)	Δλ (nm)	Fluorescence Intensity
HSA	1	226/338	112	754
	2	278/342	64	664
HSA–SMT	1	227/336	109	653
	2	279/342	63	609

**Table 3:** Secondary structure contents analysis of HSA and SMT–HSA systems.

Structure Components	α-Helix	β-Sheet	β-Turn	β-Anti
HSA	61.0%	29.4%	8.4%	1.2%
HSA–SMT	59.0%	29.5%	9.7%	1.8%

## Data Availability

Not applicable.

## Sample Availability

Samples of the compounds are not available from the authors.

## References

[1] Xie X., Huang S., Zheng J., Ouyang G. (2020). Trends in sensitive detection and rapid removal of sulfonamides: A review. J. Sep. Sci..

[2] Hashmi M.Z., Varma A. (2018). Environmental Pollution of Paddy.

[3] Bitas D., Kabir A., Locatelli M., Samanidou V. (2018). Food Sample Preparation for the Determination of Sulfonamides by High-Performance Liquid Chromatography: State-of-the-Art. Separations.

[4] Suo D., Wang P., Xiao Z., Zhang S., Zhuang H., Li Y., Su X. (2019). Multiresidue Determination of 27 Sulfonamides in Poultry Feathers and Its Application to a Sulfamethazine Pharmacokinetics Study on Laying Hen Feathers and Sulfonamide Residue Monitoring on Poultry Feathers. J. Agric. Food Chem..

[5] Correa D.A., Castillo P.M.M., Martelo R.J. (2018). Beef’s antibiotics residues determination from Arjona and Magangue municipalities in Bolivar, Colombia. Contemp. Eng. Sci..

[6] Yang Y., Qiu W., Li Y., Liu L. (2020). Antibiotic residues in poultry food in Fujian Province of China. Food Addit. Contam. Part B.

[7] Siddiqi P.M.K., Alam P., Chaturvedi S., Khan R.H. (2016). Anti-amyloidogenic behavior and interaction of Diallylsulfide with Human Serum Albumin. Int. J. Biol. Macromol..

[8] Zhang Z., Yang M., Yi J., Zhu Q., Huang C., Chen Y., Li J., Yang B., Zhao X. (2019). Comprehensive Insights into the Interactions of Two Emerging Bromophenolic DBPs with Human Serum Albumin by Multispectroscopy and Molecular Docking. ACS Omega.

[9] Su X.-Z., Chen R., Wang C.-B., Ouyang X.-L., Jiang Y., Zhu M.-Y. (2019). Astaxanthin Combine with Human Serum Albumin to Abrogate Cell Proliferation, Migration, and Drug-resistant in Human Ovarian Carcinoma SKOV3 Cells. Anti-Cancer Agents Med. Chem..

[10] Ajmal M.R., Nusrat S., Alam P., Zaidi N., Khan M.V., Zaman M., Shahein Y.E., Mahmoud M.H., Badr G., Khan R.H. (2017). Interaction of anticancer drug clofarabine with human serum albumin and human alpha-1 acid glycoprotein. Spectroscopic and molecular docking approach. J. Pharm. Biomed. Anal..

[11] Tayyab S., Sam S.E., Kabir Z., Ridzwan N.F.W., Mohamad S.B. (2019). Molecular interaction study of an anticancer drug, ponatinib with human serum albumin using spectroscopic and molecular docking methods. Spectrochim. Acta Part A Mol. Biomol. Spectrosc..

[12] Gan R., Zhao L., Sun Q., Tang P., Zhang S., Yang H., He J., Li H. (2018). Binding behavior of trelagliptin and human serum albumin: Molecular docking, dynamical simulation, and multi-spectroscopy. Spectrochim. Acta Part A Mol. Biomol. Spectrosc..

[13] Mohammadi G., Faramarzi E., Mahmoudi M., Ghobadi S., Ghiasvand A., Goicoechea H.C., Jalalvand A.R. (2018). Chemometrics-assisted investigation of interactions of Tasmar with human serum albumin at a glassy carbon disk: Application to electrochemical biosensing of electro-inactive serum albumin. J. Pharm. Biomed. Anal..

[14] Roy S., Nandi R.K., Ganai S., Majumdar K., Das T.K. (2017). Binding interaction of phosphorus heterocycles with bovine serum albumin: A biochemical study. J. Pharm. Anal..

[15] Poureshghi F., Ghandforoushan P., Safarnejad A., Soltani S. (2017). Interaction of an antiepileptic drug, lamotrigine with human serum albumin (HSA): Application of spectroscopic techniques and molecular modeling methods. J. Photochem. Photobiol. B Biol..

[16] Zhang Y., Zhang G., Zhou X., Li Y. (2013). Determination of acetamiprid partial-intercalative binding to DNA by use of spectroscopic, chemometrics, and molecular docking tech-niques. Anal. Bioanal. Chem..

[17] Cheng Z., Liu R., Jiang X. (2013). Spectroscopic studies on the interaction between tetrandrine and two serum albumins by chemometrics methods. Spectrochim. Acta A Mol. Biomol. Spectrosc..

[18] Zhang Q., Ni Y., Kokot S. (2010). Molecular spectroscopic studies on the interaction between Ractopamine and bovine serum albumin. J. Pharm. Biomed. Anal..

[19] Zhang Q., Ni Y., Kokot S. (2013). Competitive Interactions of Ionic Surfactants with Salbutamol and Bovine Serum Albumin: A Molecular Spectroscopy Study with Implications for Salbutamol in Food Analysis. J. Agric. Food Chem..

[20] Shahabadi N., Khorshidi A., Moghadam N.H. (2013). Study on the interaction of the epilepsy drug, zonisamide with human serum albumin (HSA) by spectroscopic and molecular docking techniques. Spectrochim. Acta Part A Mol. Biomol. Spectrosc..

[21] Peiris C., Gunatilake S., Mlsna T.E., Mohan D., Vithanage M. (2017). Biochar based removal of antibiotic sulfonamides and tetracyclines in aquatic environments: A critical review. Bioresour. Technol..

[22] Otagiri M., Nakamura H., Maruyama T., Imamura Y., Takadate A. (1989). Characterization of binding sites for sulfadimethoxine and its major metabolite, N4-acetylsulfadimethoxine, on human and rabbit serum albumin. Chem. Pharm. Bull..

[23] Otagiri M., Nakamura H., Imamura Y., Matsumoto U., Fleitman J., Perrin H. (1989). Effect of pH and small inorganic ions on binding of sulfadimethoxine and sulfaphenazole to human serum albumin measured by circular dichroism. Chem. Pharm. Bull..

[24] Wang J., Ma L., Zhang Y., Jiang T. (2017). Investigation of the interaction of deltamethrin (DM) with human serum albumin by multi-spectroscopic method. J. Mol. Struct..

[25] Maji A., Beg M., Mandal A.K., Das S., Jha P.K., Kumar A., Sarwar S., Hossain M., Chakrabarti P. (2017). Spectroscopic interaction study of human serum albumin and human hemoglobin with Mersilea quadrifolia leaves extract mediated silver nanoparticles having antibacterial and anticancer activity. J. Mol. Struct..

[26] Condurache N.N., Aprodu I., Grigore-Gurgu L., Petre B.A., Enachi E., Râpeanu G., Bahrim G.E., Stănciuc N. (2020). Fluorescence spectroscopy and molecular modeling of anthocyanins binding to bovine lactoferrin peptides. Food Chem..

[27] Zu F., Yan F., Bai Z., Xu J., Wang Y., Huang Y., Zhou X. (2017). The quenching of the fluorescence of carbon dots: A review on mechanisms and applications. Mikrochim. Acta.

[28] Yang H., Zeng Q., He Z., Wu D., Li H. (2020). Interaction of novel Aurora kinase inhibitor MK-0457 with human serum albumin: Insights into the dynamic behavior, binding mechanism, conformation and esterase activity of human serum albumin. J. Pharm. Biomed. Anal..

[29] Ross P.D., Subramanian S. (1981). Thermodynamics of protein association reactions: Forces contributing to stability. Biochemistry.

[30] Ghobadi S., Ashrafi-Kooshk M.R., Mahdiuni H., Khodarahmi R. (2018). Enhancement of intrinsic fluorescence of human carbonic anhydrase II upon topiramate binding: Some evidence for drug-induced molecular contraction of the protein. Int. J. Biol. Macromol..

[31] Zhang M., Chai Y., Han B. (2019). Mechanistic and Conformational Studies on the Interaction Between Myriocin and Human Serum Albumin by Fluorescence Spectroscopy and Molecular Docking. J. Solut. Chem..

[32] Xu L., Liu Z., Liao T., Tuo X. (2019). Probing the interaction between levamlodipine and hemoglobin based on spectroscopic and molecular docking methods. Spectrochim. Acta Part A Mol. Biomol. Spectrosc..

[33] Ma X., Yan J., Xu K., Guo L., Li H. (2016). Binding mechanism of trans-N-caffeoyltyramine and human serum albumin: Investigation by multi-spectroscopy and docking simulation. Bioorg. Chem..

[34] Liao T., Zhang Y., Huang X., Jiang Z., Tuo X. (2021). Multi-spectroscopic and molecular docking studies of human serum albumin interactions with sulfametoxydiazine and sulfamonomethoxine. Spectrochim. Acta Part A Mol. Biomol. Spectrosc..

[35] Ren C., Xiong W., Li B. (2019). Binding interaction between β-conglycinin/glycinin and cyanidin-3-O-glucoside in acidic media assessed by multi-spectroscopic and thermodynamic techniques. Int. J. Biol. Macromol..

